# Social deficits mirror delayed cerebrovascular dysfunction after traumatic brain injury

**DOI:** 10.1186/s40478-024-01840-w

**Published:** 2024-08-07

**Authors:** Aditya Singh, Steven Gong, Anh Vu, Scott Li, Andre Obenaus

**Affiliations:** 1 Department of Pediatrics, School of Medicine, University of California Irvine, Hewitt Hall Rm. 2066, Irvine, CA 92697 USA; 2 Division of Biomedical Sciences, 206 SOM Research Bldg, University of California Riverside, Riverside, CA 92521 USA; 3 Department of Neurology, The Lundquist Institute for Biomedical Innovation at Harbor-UCLA, 120 Walter P Martin Research Center, Torrance, California 90502 USA; 4 Department of Neurosurgery, University of California Los Angeles, Los Angeles, California 90095 USA

**Keywords:** Social isolation behavior, Perfusion weighted MRI, Vascular networks, Cerebral blood flow, Open field behavior, Long-term effects of brain injury

## Abstract

Traumatic brain injury (TBI) survivors face debilitating long-term psychosocial consequences, including social isolation and depression. TBI modifies neurovascular physiology and behavior but the chronic physiological implications of altered brain perfusion on social interactions are unknown. Adult C57/BL6 male mice received a moderate cortical TBI, and social behaviors were assessed at baseline, 3-, 7-, 14-, 30-, and 60-days post injury (dpi). Magnetic resonance imaging (MRI, 9.4T) using dynamic susceptibility contrast perfusion weighted MRI were acquired. At 60dpi mice underwent histological angioarchitectural mapping. Analysis utilized standardized protocols followed by cross-correlation metrics. Social behavior deficits at 60dpi emerged as reduced interactions with a familiar cage-mate (partner) that mirrored significant reductions in cerebral blood flow (CBF) at 60dpi. CBF perturbations were dynamic temporally and across brain regions including regions known to regulate social behavior such as hippocampus, hypothalamus, and rhinal cortex. Social isolation in TBI-mice emerged with a significant decline in preference to spend time with a cage mate. Cortical vascular density was also reduced corroborating the decline in brain perfusion and social interactions. Thus, the late emergence of social interaction deficits mirrored the reduced vascular density and CBF in regions known to be involved in social behaviors. Vascular morphology and function improved prior to the late decrements in social function and our correlations strongly implicate a linkage between vascular density, cerebral perfusion, and social interactions. Our study provides a clinically relevant timeline of alterations in social deficits alongside functional vascular recovery that can guide future therapeutics.

**Supplementary Information:**

The online version contains supplementary material available at 10.1186/s40478-024-01840-w.

## Introduction

Traumatic brain injury (TBI) afflicts an estimated 70 million people across the world annually with both age and sex as important determinants of severity and outcomes [1, 2]. Mild to moderate TBI are the most common type of injuries among civilians, professional sports personnel, and military service members [3, 4]. Behavioral, physiological and psychosocial deficits persist for extended periods after the initial injury and include depression, post-traumatic stress, sleep, Alzheimer’s disease, and dementia related disorders amongst others [5–8]. Despite advances in our understanding of the pathophysiological processes underlying TBI, there is a gap in the linkage between behavioral outcomes and cerebrovascular alterations following TBI.

Social interactions are impaired after TBI with reductions in interpersonal communication, [9] time with friends and families, [10, 11] and is evident in children [12, 13] and adults [14, 15]. Social behavior is also diminished in adult mice after a single [16, 17] or repeated-mild TBI, [18, 19] and in pediatric models of TBI [20]. Social isolation in rodents reflects increased neuropathology after TBI, [21] however, increased social interaction post-injury is known to facilitate recovery [22]. Lacking are studies that examine how TBI modifies sociability and its relationship to disrupted cerebrovascular morphology and function.

We and others have reported acute vascular alterations following TBI spanning chronically reduced microvasculature, [23–25] and global reductions in cerebrovascular reactivity and tone [26–28]. TBI induced changes in the vasculature are associated with motor and cognitive behavioral deficits, [29, 30] while others have reported no change at long-term after injury [31]. Autism spectrum (ASD) subjects, which manifest deficits in social interactions, exhibited CBF reductions that correlated with the severity of behavioral alterations [32]. Further, intranasal treatment with oxytocin increased blood flow across social processing brain regions [33] and monitoring vascular metrics has been proposed as a biomarker for social interactions [34]. 

To address the paucity of knowledge linking physiological CBF, underlying angioarchitecture, and social behavior deficits after moderate TBI, we undertook a longitudinal study in adult male mice. Specifically, we tested the hypothesis that long-term cerebrovascular deficits facilitate social dysfunction. We report that temporal vascular flow and morphology initially recover but ultimately decline by 2 months post TBI which are mirrored by social interaction deficits. Our novel study provides the basis for future preclinical and clinical interventional studies targeting social psychopathologies following acquired moderate TBI.

## Materials and methods

### Animals

**Fig. 1:**
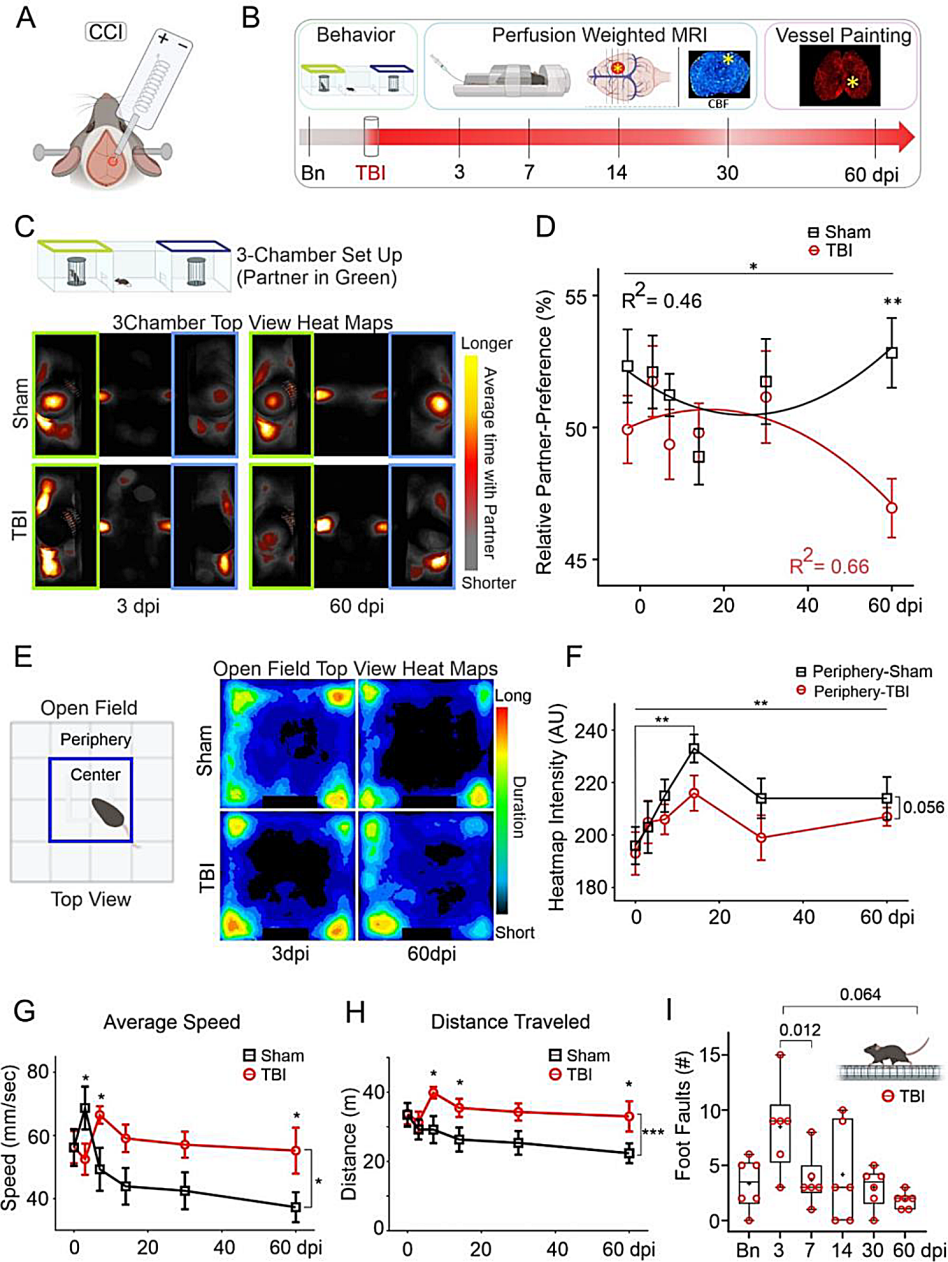
Chronic social and exploratory behavior changes following TBI. (**A**) Cortical contusion injury (CCI) in adult male mice centered at somatosensory and motor cortices. (**B**) Behavioral and MRI experimental timeline with vessel painting at 60 days post injury (dpi). (**C**) Heat maps of 3-chamber social behavior utilized a known cage-mate mouse (partner) and illustrate increased cumulative time spent by sham (top) and TBI mice (bottom row) at 3 and 60dpi (**D**) Relative partner-preference (RPP) is significantly reduced at 60dpi in TBI (red circles) relative to sham mice (black squares) (2wANOVA - Injury factor - *F*(1,60) = 5.02, *p = 0.029, Tukey’s post-hoc 60dpi. sham vs. TBI - ** *p* = 0.002). (**E**) Open-field arena schematic (left) with center and periphery zones. Heatmap of average time spent at 3- and 60dpi for sham (top row) and TBI animals (bottom row) illustrates increased center time after TBI. (**F**) Sham mice (black, squares) spent significantly more time in periphery at 14dpi vs. baseline (2wANOVA: *F*(5,61) = 0.006, Tukey’s post hoc **p = 0.008) but reduced time in TBI mice (Injury factor, 2wANOVA, *F*(1, 61) = 3.80, *p* = 0.056). (**G**) TBI mice in open field exhibited increased average speed compared to shams (2wANOVA, injury factor, *F*(1, 61) = 6.44, *p = 0.014). (**H**) Total distance travelled in open field was also significantly increased in TBI mice vs. shams (2wANOVA, injury factor, *F*(1, 61) = 14.4, ***p = 0.0003) at 7, 14 and 60dpi (*p < 0.05). (**I**) Sensorimotor tests in TBI mice at 3dpi exhibited increased foot-faults with modest longitudinal recovery. (dpi - days post injury, CCI - Cortical contusion injury, TBI - Traumatic brain injury, partner - cage mate mouse, CBF - cerebral blood flow, Bn - baseline, bright asterisk on coronal and axial view of the brain - TBI impact site)

All experiments were conducted using ARRIVE guidelines and animal use was approved by the University of California Irvine Animal Care and Use Committee. Adult C57/B6 male mice (JAX#000664, 2-3months old) from Jackson Laboratory were group housed (3/cage) with 12 h light/dark cycle in ventilated cages and acclimated for a minimum of 7d after arrival. Male C57BL/6 mice were randomly assigned to either sham surgery (*n* = 11) or a moderate TBI (*n* = 10) followed by longitudinal behavior and perfusion MRI across a 60d post injury (dpi) time course (Fig. 1A, B). Animal numbers were based on literature and pilot experiments, and subsequent statistical power estimations. A subset of sham and TBI mice (*n* = 6/group) were relegated for foot-fault behavior. Two of ten TBI mice died at 30dpi. Sham and TBI mice maintained similar weight profiles (Supplementary Fig. 1).

### Traumatic brain injury (TBI)

TBI with controlled cortical impact (CCI, Fig. 1A) was induced as previously reported [25, 35] and detailed in the supplementary materials. Briefly, anesthetized (isoflurane 1–3%) mice were maintained at 37^o^C and then placed in a stereotaxic device. Under aseptic conditions, a scalp incision was made, underlying connective tissue retracted and a 5 mm craniotomy (bregma AP − 1.25 cm, ML + 1.25 cm) was performed to expose the brain. A 1.5 mm impactor tip was zeroed to pial surface and electromagnetic impactor was discharged (Leica, NeuroscienceTools, O’Fallon) with the following parameters: 1 mm depth, 200ms dwell-time, speed 5 m/s. Extravascular bleeding was immediately wicked away and the skin was sutured closed without replacing the bone. Buprenorphine (100ng/g body weight, intramuscular) was injected, and mice were returned to a warmed chamber until ambulatory. Sham mice were exposed to the identical anesthesia duration but did not experience a craniotomy [23].

### Behavioral paradigms

Mice were carefully handled to habituate with experimenter 1–2 days before testing [36]. Behavioral testing sequentially included foot-fault (FF), open-field (OF), 3-chamber social preference (3Ch) tests prior to each magnetic resonance imaging (MRI) session for a subgroup of animals (*n* = 12 for baseline, 3, 7, 14 and 30dpi, *n* = 16 at 60dpi). Mice in their home cages were acclimatized to the behavior room for 5–10 min before onset of testing. Mice were allowed to rest for 10 min in their home cage between testing paradigms. All apparatus were disinfected before and after each mouse. Extended behavioral details are delineated in the Supplementary materials.

### Foot-fault (FF)

Mice were individually placed at one end of a grid (47.5 × 29.5 cm, 25 beams, 1.5 cm apart) and allowed to walk freely until they reached their home cage at the other end of the grid or 120 s, whichever occurred first. Foot slips through the grid were manually counted by two blinded experimenters. Only *n* = 6 sham and *n* = 6 TBI mice were tested for foot-fault.

### Open field (OF)

Mice were placed in the center of an open field arena (30 × 30cm^2^) and allowed free exploration for 10 min (top view webcam recording).

### 3-Chamber social preference (3Ch)

Our 3Ch test utilized a known cage-mate (partner) being placed inside a wireframe enclosure in one of the peripheral chambers and alternated between each session [37]. The peripheral chambers were connected to the central chamber (15.5 × 28.50cm^2^) with manual sliding doors. The test mouse was placed in the central chamber with closed doors for 5 min and then doors were opened to allow free access to both peripheral chambers for 10 min. The behavior was video recorded for offline analysis.

### Behavior analyses

All semi-automated image analyses or manual scoring were blinded to injury condition. We utilized multiple software, including Fiji [38] for OF and 3Ch, and AnimalTA [39] for OF animal tracking to estimate speed and distance. Videos were cropped to isolate the identical regions of interest (ROIs) for OF and 3Ch (Supplementary Fig. 2). FIJI’s image adjust algorithm was used with automated minimum threshold for animal (red) and background (dark) detection, to generate binary masks. Thresholded stacks were averaged as heat-maps (Fiji), with pixel-intensity representing time [40] allowing measurement of time spent in partner vs. no-partner chambers, termed relative partner-preference (RPP). Manual scored behavior utilized BORIS [41] to derive, (a) absolute partner-interaction time (API) defined as total time the test mouse was facing the cylindrical enclosure with the partner mouse, and (b) relative partner-interaction time (RPI), defined as the ratio of time spent interacting with (pointed towards) partner enclosure vs. total interaction time across both partner and non-partner enclosures.

### MRI

In vivo longitudinal MRI was performed (Fig. 1B) at baseline and after TBI induction (3, 7, 14, 30, 60dpi) on a horizontal 30 cm bore, 9.4Tesla MR scanner (Bruker Avance) equipped with a 72 mm diameter volume excitation RF coil. Our perfusion weighted imaging (PWI) MRI methods are published [42] and described in more detail in the supplementary materials. Succinctly, mice were anesthetized (2% isoflurane) and tail veins were cannulated to facilitate contrast injection (0.1mmol/kg Gadoterate Meglumine diluted with sterile saline, Dotarem, Guerbet, Princeton, NJ). Sham or TBI mice were then pseudo-randomly placed in MRI and the following sequences were acquired: T2-weighted (T2), T1-weighted images (T1), PWI during which Gd was infused (1ul/g body weight), and susceptibility-weighted MRI (Supplementary Table 1 for MRI sequence details).

### MRI image analysis

Detailed MRI processing methods are reported in Supplementary materials. PWI MRI was processed using Jim software (V9.1, Xinapse Systems Ltd, Essex, UK) using the Brain Perfusion tool to automatically derive the arterial input function (AIF) curves which were manually reviewed for typical AIF profiles. AIF curves from each sham mouse across all six time points were averaged for a group average AIF [43] and used to calculate cerebral blood flow (CBF, ml/100 g-tissue/min) and cerebral blood volume (CBV, %tissue) [44]. TBI animals used individual AIF curves to calculate CBF and CBV to account for variability due to injury. In Jim software an in-house mouse atlas was applied to CBF and CBV parametric maps, values were extracted and summarized in Excel. MRI analyses were performed blind with respect to the behavior data.

### Vessel painting and analyses

To visualize cortical angioarchitecture we utilized our vessel painting protocol as previously reported (see supplementary materials) [35]. Briefly, 1,1’-Dioctadecyl-3,3,3’,3’-Tetramethylindocarbocyanine Perchlorate (DiI, D282, Invitrogen, Carlsbad) was delivered via intracardiac injection prior to transcardial fixative perfusion at 60dpi and brains were extracted after 24 h post fixation, rinsed in 4% PFA for 24 h and stored in PBS + 0.02% sodium azide until microscopic acquisition. All animals had successful staining of the cortical vasculature (*n* = 8 TBI, *n* = 11 sham).

Vascular image acquisition and analysis protocols have been previously published [23, 35]. Briefly, the bilateral axial cortices were imaged at 2X magnification using an epifluorescent wide-field microscopy ( BZ-X810, Keyence Corp., Osaka) and 10x magnification images of the middle cerebral artery (MCA) of the ipsilateral hemisphere. Classical vascular analysis for vessel density, junctional branch points, total end points, and average and total vessel length were obtained using the Fiji plugin, Angiotool [45]. Analysis focused on lesion and perilesional regions (see Fig. 4A). Fractal analyses for vascular complexity was also performed using Fiji Fraclac plugin to obtain local fractal dimensions (LFD).

### Statistics

Behavioral indices, MRI, and vessel painting derived values were imported into MS-Excel and MRI data were filtered for outliers using interquartile ranges. Data was assumed to be normally distributed and confirmed during statistical testing. Any mouse that exhibited regional data outliers > 30% (CBF or CBV smaller or larger than 1.5 times the IQR) were excluded for calculating averages (Supplementary Table 2). Accordingly, statistical tests, Pearson’s correlation coefficient estimations, and plotting were performed using MS-Excel or Prism 9.0 (GraphPad, San Diego). 3D-CBF plot was generated using custom python script (Plotly.com). Scatter plots used box and whisker graphs with mean and error bars (minimum to maximum values) where the box bounding represents 25th and 75th percentiles. In line graphs error bars are plotted as standard error of mean (SEM). Two-way ANOVA (2wANOVA) with Tukey’s *Post-hoc* test was used for statistical comparisons, unless specified otherwise. Statistical significance was noted at *p < 0.05, **p < 0.01, or ***p < 0.001, with trending as *p* < 0.1.

## Results

### Long-term reduction in social behavior after TBI

Animals underwent longitudinal evaluation following a CCI (Fig. 1A, B) alongside social behavior using a 3Ch paradigm with cage-mate mice. Similar relative partner-preference (RPP) at 3dpi with dramatic reductions at 60dpi (Fig. 1C, Supplementary Fig. 3A-D). Semi-automated quantitative image analyses confirmed a significant RPP decrease in cage-mate interaction (2wANOVA, Injury: *F*(1,60) = 5.02, *p = 0.029) with significant post-hoc reductions in TBI animals at 60dpi (Adj. **p = 0.002) (Fig. 1D, Supplementary Fig. 4). Manual video scoring confirmed similar RPP profiles (Supplementary Fig. 3A-D). The social deficits were independent of motor deficits, as evident in FF and OF tests, confirming comparable speeds with higher exploratory drive likely reflecting increased risk-taking behavior in TBI-mice vs. shams (Fig. 1E-H).

### Increased exploratory behavior after TBI

Increased risk-tasking in TBI mice was evident with significantly reduced OF periphery activity as early as 14dpi through to 60dpi relative to shams (Fig. 1E, F) (2wANOVA, Injury, *F*(1,61) = 3.80, *p* = 0.056; Timepoints, *F*(5,61) = 3.64, **p = 0.006). Post-hoc, sham mice spent more time in periphery at 14dpi compared to baseline (**p = 0.008). Ratio of time spent in center/periphery found no significant differences (Supplementary Fig. 3F).

TBI mice exhibited higher speeds and distance travelled from 7dpi onwards across all the timepoints with significant ‘time X injury’ interactions for speed (Fig. 1G-H, Supplementary Fig. 3E-H); (Fig. 1G, average speed, 2wANOVA, Injury, *F*(1,61) = 6.44, *p = 0.014; Timepoint, *F*(5,61) = 1.91, *p* = 0.1, Interaction *F*(5,61) = 2.99, *p = 0.018); (Fig. 1H, total distance, 2wANOVA, Injury, *F*(1,61) = 14.4, ***p = 0.0003; Timepoint, *F*(5,61) = 1.28, *p* = 0.28, Interaction *F*(5,61) = 1.01, *p* = 0.42). TBI animals had significantly reduced speed at 3dpi (Adj.*p = 0.045) but elevated at 7dpi (Adj. *p = 0.027) and 60dpi (Adj.*p = 0.027) vs. sham mice. Distance travelled was elevated at 7dpi (Adj.*p = 0.017), 14dpi (Adj. *p = 0.048), and 60dpi (Adj.*p = 0.022) for TBI vs. sham mice (Fig. 1H).

### Early sensorimotor deficits post-TBI recover with time

Sensorimotor failures assessed using FF testing were increased in TBI compared to sham mice at 3dpi (Supplementary Figs. 5, 1wANOVA, *F* = 3.99, *p* < 0.001, *Post hoc* 3dpi TBI vs. Sham, **p = 0.001), with return to baseline between 7-60dpi (Fig. 1I, rmANOVA, *F*(4.28, 11.8) = 4.28, *p = 0.035, post-hoc: 3- vs. 7dpi *p = 0.012, 3- vs. 60dpi *p* = 0.064).

### CBF dysfunction mirrors social behavior deficits

**Fig. 2:**
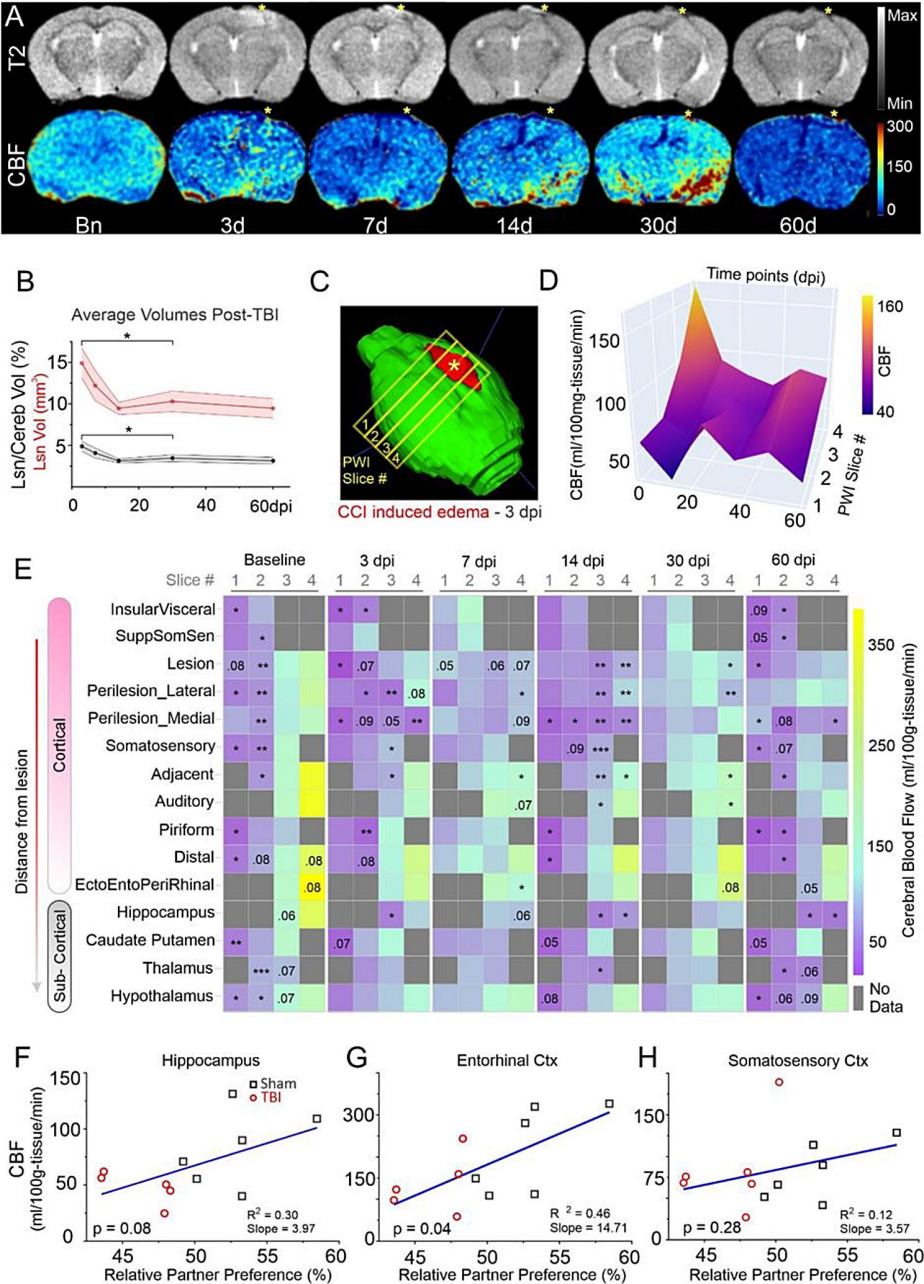
Cerebral blood flow (CBF) recovers but declines at 60dpi. (**A**) Representative T2-weighted anatomical MR images and CBF maps (ml/100 g-tissue/min) from the same TBI mouse illustrates transient decrements, recovery and then followed by precipitous decline at 60dpi. Edema at 3dpi at the impact site (asterisk) resolves and is followed by moderate tissue loss 14-60dpi. (**B**) Lesion volume (mm^3^, red; % brain volume, black) edema increases and stabilizes after edema resolution (1wANOVA – Lesion Volume, *F*(2.17, 18.4) = 5.43, *Geisser-Greenhouse’s *=0.541, *p = 0.013, *Tukey’s* post-hoc 3- vs. 30dpi *p = 0.47; Lesion/Cerebrum volume, *F*(2.18, 18.5) = 5.37, *Geisser-Greenhouse’s *=0.544, *p = 0.013, *Tukey’s* post-hoc 3- vs. 30dpi *p < 0.05). (**C**) Brain 3D-reconstruction in a TBI mouse (3dpi) illustrates edematous lesion (red). PWI MRI data were collected from four 1 mm thick coronal slices. (**D**) Temporal evolution (Baseline-60dpi) of CBF at lesion site across antero-posterior slices with acute reductions at 3dpi, recovery followed by declines at 60dpi. (**E**) CBF heatmap depicting longitudinal CBF changes for each slice (columns) with brain regions (rows) sorted by distance from TBI impact site. Statistical significance (t-test TBI vs. Sham) is noted (* *p* < 0.05, ** *p* < 0.01, *** *p* < 0.001) as are trending *p*-values. Reduced CBF was evident in anterior slices but increased in posterior slices distant from TBI site. **(F-H)** Correlations between 60dpi CBF and relative partner preference (RPP) in sham and TBI mice in social exploration related brain regions (dorsal hippocampus *p* = 0.08, *R*^2^ = 0.30 (**F**), entorhinal cortex *p* = 0.04, *R*^2^ = 0.46 (**G**) and somatosensory cortex (*p* = 0.28, *R*^2^ = 0.12, (**H**))

Structural T2WI in TBI mice exhibited early edema which resolved over time (3-7dpi) with subsequent cortical thinning at the impact site (14-60dpi; Fig. 2A). Lesion volumes were initially elevated during the edematous phase and then stabilized to ~ 10mm^3^ or ~ 4% of brain volume (Fig. 2B, C). CBF exhibited regional and global declines that gradually recovered over the initial 30dpi, but then steeply declined at 60dpi (Fig. 2A, D). CBF at the cortical impact site in TBI mice had acute reductions at 3dpi, modest recovery during 7-30dpi, followed by precipitous CBF declines at 60dpi (Fig. 2D, mixed effect 1wANOVA, slice#1: *F*(2.993, 24.55) = 3.71, *p = 0.02, 3- vs. 7dpi *p = 0.036, and 3- vs. 30dpi *p = 0.046; slice#4: *F*(2.66, 23.38) = 5.20, Tukey’s post-hoc: Bn vs. 7dpi *p = 0.031, Bn vs. 14dpi ***p < 0.001, Bn vs. 30dpi *p = 0.036, and Bn vs. 60dpi **p = 0.006). TBI induced CBF perturbations extended beyond the impact site to adjacent and distant ipsilateral cortical and subcortical regions (Fig. 2A, E) where CBF heatmaps highlight the regional multiphasic nature of physiological recovery. Like the injury site profile (Fig. 2D), distant regions reflected an initial CBF decline at 3dpi, transient recovery at 7-30dpi with a subsequent decline at 60dpi (Fig. 2E). We then examined the relationship between social behavior (RPP) and CBF at 60dpi which demonstrated positive correlations in regions involved in exploratory and social behavior (Fig. 2F-H). Hippocampal CBF was trending positively correlated to RPP (Fig. 2F, *p* = 0.08, *R*^*2*^ = 0.30) and was significantly correlated in the entorhinal cortex (Fig. 2G, *p = 0.04, *R*^*2*^ = 0.30), but not in somatosensory cortex (Fig. 2H, *p* = 0.28, *R*^*2*^ = 0.12). Thus, TBI elicits a dynamic profile of tentative recovery followed by regional reductions that correlated to indices of social isolation.

### Cortical and sub-cortical progression of CBF dynamics post-TBI

**Fig. 3:**
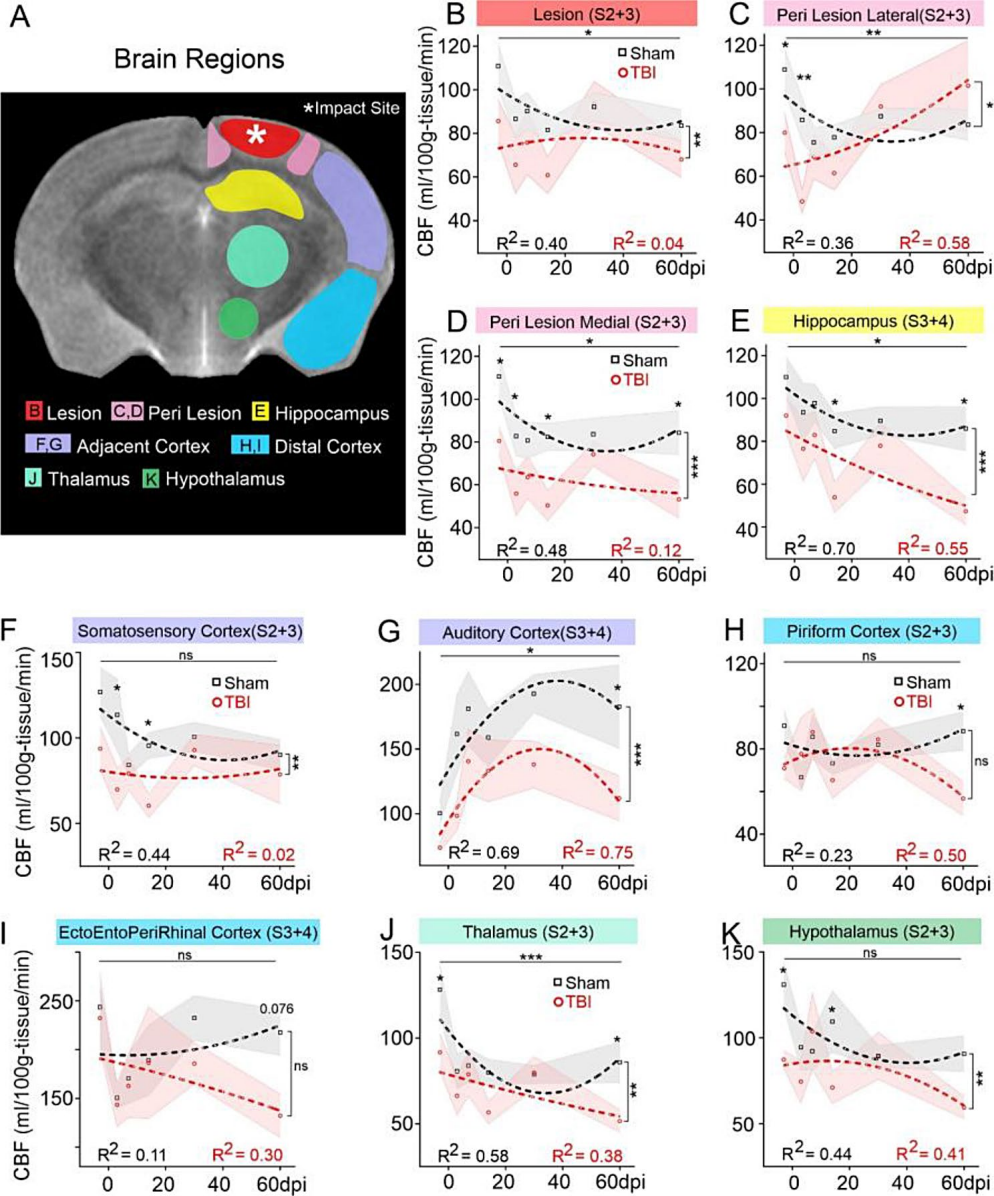
Longitudinal CBF dynamics in brain regions. (**A**) Representative regions of interest (ROIs) on a coronal MRI. (**B**) CBF at lesion cortex shows overall significant difference across both timepoints (6-sessions 0-60dpi, *F*(5,105) = 2.51, *p = 0.035) and injury condition (Sham vs. TBI, *F*(1,105) = 8.36, **p = 0.005). (**C**) Increased CBF in lateral peri-lesional cortex of TBI mice was significant across timepoints (*F*(5,106) = 3.77, **p = 0.003) and injury conditions (*F*(1,106) = 4.47, *p = 0.037) and interactions (*F*(5,106) = 2.51, *p = 0.034). (**D**) Medial peri-lesion cortex CBF was significantly reduced across time (*F*(5,107) = 3.06, *p = 0.013) and injury condition (*F*(1,107) = 23.0, ***p < 0.001, interaction–ns). (**E**) CBF profile in dorsal hippocampus was significantly different across time (*F*(5,107) = 3.10, *p = 0.012) and injury condition (*F*(1,107) = 14.3, ***p < 0.001, interaction–ns). (**F**) Somatosensory cortex showed stable CBF across all timepoints (*F*(5,106) = 1.88, *p* = 0.104, ns) with higher overall longitudinal trend for shams compared to TBI animals (injury factor, *F*(1,106) = 10.4, **p = 0.002, interaction–ns). (**G**) Auditory cortex profiles were significantly different across time (*F*(5,107) = 3.16, *p = 0.011) and injury condition (*F*(1,107) = 12.7, ***p < 0.001, interaction–ns). (**H**) Piriform cortex reported stable CBF profiles across timepoints (*F*(5,105) = 1.20, *p* = 0.316, ns) and injury condition (*F*(1,105) = 1.86, *p* = 0.175, ns and post-hoc comparisons identified significantly lower CBF at 60dpi in TBI vs. sham mice(*p = 0.025). (**I**) Rhinal cortices (ento, ecto, peri) showed an overall similar CBF trend across time (*F*(5,106) = 2.11, *p* = 0.069, trending) and injury condition (*F*(1,106) = 2.13, *p* = 0.147, ns). Post-hoc comparison found a trending decline at 60dpi for TBI vs. sham (*p* = 0.076). **(J)** CBF in thalamus was significantly different across timepoints (*F*(5,104) = 4.57, ***p < 0.001) and injury condition (*F*(1,104) = 10.0, **p = 0.002, interaction–ns). **(K)** Hypothalamus found stable CBF profiles across time (*F*(5,103) = 1.56, *p* = 0.177) with significantly different perfusion across injury conditions (*F*(1,103) = 7.73, **p = 0.006). (numbers in regional titles denote the PWI slice data)

We next investigated regional CBF profiles as a function of distance from the impact site (Fig. 3A). The lesion site CBF was significantly lower across the 60dpi epoch, reflecting protracted neurovascular damage after TBI (Fig. 3B, Injury, *F*(1,105) = 8.36, **p = 0.005, Timepoints, *F*(5,105) = 2.51, *p = 0.035). After an initial decline, lateral perilesional (somatosensory) cortex CBF progressively increased above shams (Fig. 3C, Injury, *F*(1,106) = 4.47, *p = 0.037, Timepoints, *F*(5,106) = 3.77, **p = 0.003). *Post hoc*, CBF at 3dpi was acutely reduced after injury (**p = 0.004). Longitudinally for TBI animals, CBF at 3dpi was lower compared to 30dpi (*p = 0.026) and 60dpi (**p = 0.003), and 14dpi CBF was lower vs. 60dpi (*p = 0.042) in TBI animals, demonstrating a multiphasic pattern with long-term increment after initial decline. Notably, the recovery by 30dpi was reflected as similar CBF in sham and TBI animals. Medial perilesional (retrosplenial) cortex also had significant reductions in CBF across time (Fig. 3D, Injury, *F*(1,107) = 23.0, ***p < 0.001, Timepoints, *F*(5,107) = 3.06, *p = 0.013). CBF in TBI mice was reduced relative to shams at 3 (*p = 0.028), 14 (*p = 0.011), and 60dpi (*p = 0.021).

Hippocampus exhibited reduced CBF across the 60dpi period in TBI mice (Fig. 3E, Injury, *F*(1,106) = 14.3, ***p < 0.001, Timepoints, *F*(5,106) = 3.10, *p = 0.012) with significant reductions compared to shams at 14 (*p = 0.037) and 60dpi (*p = 0.016). CBF within the cortical regions adjacent to injury (somatosensory, auditory) showed complementary temporal dynamics. In somatosensory cortex TBI mice had reduced CBF spanning the entire experimental period whilst shams had temporal reductions but were not significantly different albeit there was a significant group effect (Fig. 3F, Injury, *F*(1,106) = 10.40, **p = 0.002, Timepoints, *F*(5,106) = 1.88, *p* = 0.104). TBI CBF was significantly lower relative to shams at 3 (*p = 0.012) and 14dpi (*p = 0.04). In the adjacent auditory cortex but more distant from TBI site, CBF increased over the first 30dpi but then declined at 60dpi (Fig. 3G, Injury, *F*(1,104) = 9.31, **p = 0.003, Timepoints, *F*(5,104) = 2.63, *p = 0.028) with significantly reduced CBF in TBI mice only at 60dpi (*p = 0.035).

Even more distant from the TBI site, two ventrolateral cortical regions (piriform, ento-ecto-peri-rhinal) exhibited similar declines at 60dpi (Fig. 3H, I), with no significant injury vs. time interactions. Piriform cortex was significantly reduced at 60dpi (Post-hoc, Sham vs. TBI *p = 0.025) with trending reductions in the rhinal cortices (ento, ecto, peri) at 60dpi (Post-hoc, Sham vs. TBI, *p* = 0.076).

Subcortical regions such as thalamus and hypothalamus are involved in social behavior (Fig. 3J, K) [46]. Sham animals showed higher thalamic CBF than TBI mice at baseline (Fig. 3J, Injury, *F*(1,104) = 10.0, **p = 0.002, Timepoints, *F*(5,104) = 4.57, ***p < 0.001, *Post hoc*, baseline *p = 0.013, 60dpi *p = 0.027). Hypothalamus had no temporally significant CBF changes (*F*(5,103) = 1.56, *p* = 0.177) but significant differences across injury conditions (*F*(1,103) = 7.73, **p = 0.006) with TBI mice having lower CBF at baseline (*p = 0.017) and 14dpi (*p = 0.032) compared to shams.

### Reduced cortical angioarchitecture coincides with social behavior decrements at 60dpi

**Fig. 4:**
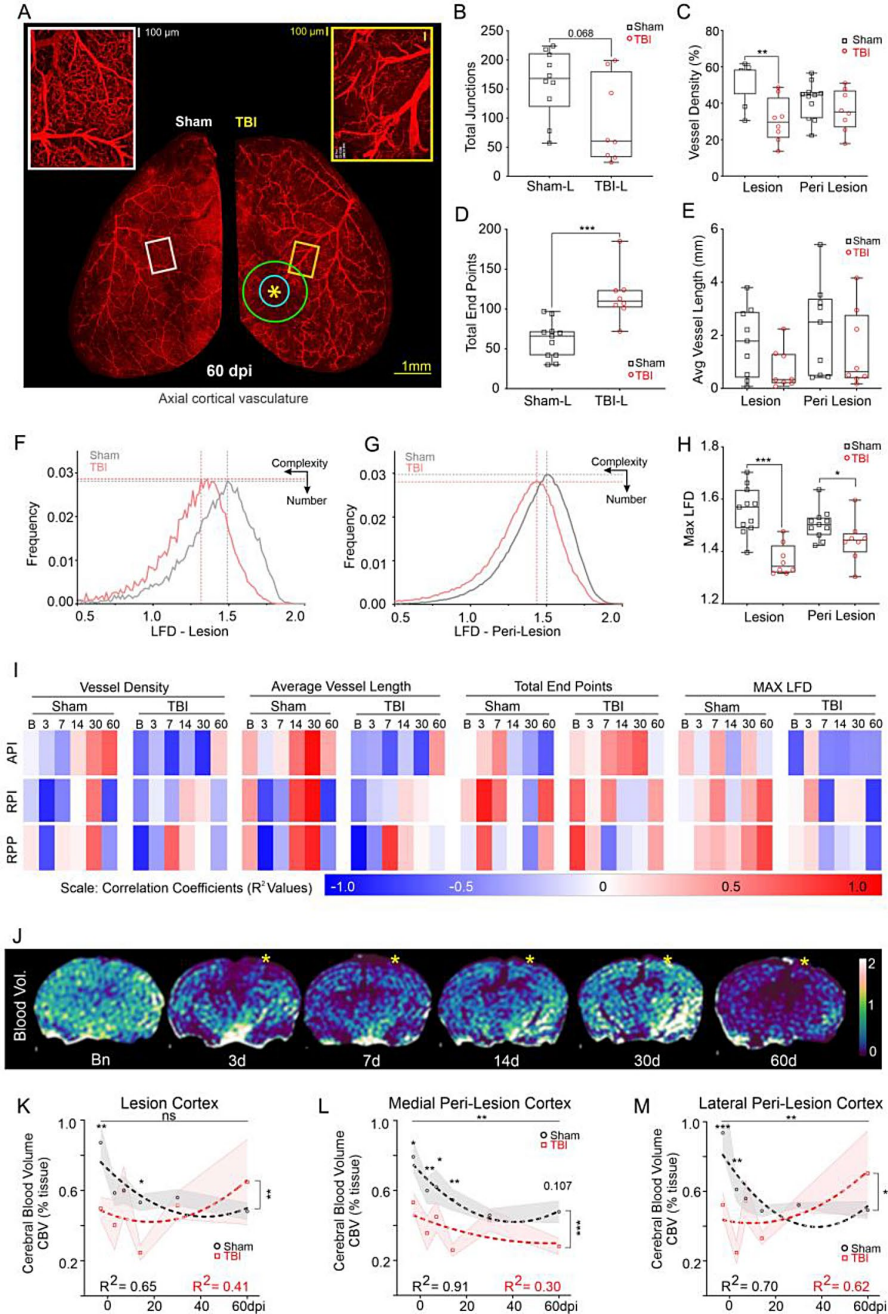
Vascular networks at 60dpi are perturbed. (**A**) Axial cerebral vasculature is reduced in TBI (right) compared to sham (left) mice in vessel networks encompassing the middle cerebral artery (MCA). Green circle = peri lesion ROI, blue circle = lesion ROI, yellow asterisk = impact site. (**B**) Number of vessel junctions were reduced within the TBI lesion (*p* = 0.068) (red circles) compared to shams (black squares). (**C**) Lesion site vessel density in TBI mice was significantly (**p = 0.002) reduced compared to shams but not in peri-lesional cortex. (**D**) Vessel end points were significantly increased at TBI lesion site compared to shams (***p = 0.0001). (**E**) Average vessel length was unaltered between sham and TBI mice. **(F**,** G)** Fractal analysis identified a leftward shift (reduced vessel complexity) in local fractal dimension (LFD) histograms in the lesion (**F**) and in peri-lesion (**G**) sites. (**H**) Maximum local fractal dimension (LFD) was significantly reduced in lesion (***p = 0.0001) and peri-lesion sites (*p = 0.047) for TBI mice relative to shams (B-H: 2-tailed-Mann-Whitney-Test). (**I**) Vascular network parameters at 60dpi were correlated to temporal social outcomes across sham and TBI mice, with opposite correlations between groups. Top row – Absolute partner-interaction-time (API), middle row – Relative partner-interaction time (RPI), bottom row – Relative partner-preference (RPP). **(J)** Transient decrements in cerebral blood volume (CBV, % tissue) over the first 14dpi recovers by 30dpi but is followed by a dramatic 60dpi decrease. **(K)** Lesion cortical CBV was low initially but slowly increased after 30dpi (*F*(5,104) = 2.20, *p* = 0.059). CBV profiles were significantly different across time but not injury condition (sham vs. TBI, *F*(1,104) = 7.27, **p = 0.008, interaction–ns). **(L)** Medial peri-lesion cortex had significantly reduced CBV across timepoints (*F*(5,102) = 3.76, **p = 0.004) and injury condition (*F*(1,102) = 24.2, ***p < 0.001, interaction–ns). **(M)** CBV in lateral peri-lesion cortex has a similar trajectory as lesion cortex with significant differences across temporal (*F*(5,101) = 3.68, **p = 0.004) and injury condition (*F*(1,101) = 6.87, *p = 0.010), and interactions (*F*(5,101) = 3.54, **p = 0.005). TBI animals had higher CBV at 60dpi (**p = 0.002)

We previously reported recovery of cortical vasculature by 30dpi after TBI [25]. Surprisingly, at 60dpi we observed broad disturbances to vascular morphology that mirrored CBF reductions (Fig. 4A). At TBI site (lesion), vessel junctions had a trending reduction (*p* = 0.068) in TBI mice compared to shams (Fig. 4B), while vessel density was significantly decreased (*p* = 0.002, Fig. 4C) accompanied by significantly increased vascular endpoints (*p* = 0.0001, Fig. 4D). Average vessel length was unaltered between sham and TBI mice (Fig. 4E). Fractal analysis confirmed reduced vascular complexity that mirrored reduced vessel density (Fig. 4F-H). Maximum local fractal dimension (LFD) was significantly reduced at the lesion (*p* = 0.0001) and the peri-lesion sites (*p* = 0.047) compared to shams (Fig. 4H-tailed-Mann-Whitney Test). Thus, impaired angioarchitecture at 60dpi provides an anatomical basis for our observed physiological and behavioral decrements.

Further linkage between vascular anatomy and longitudinal social behavior was assessed via correlations. Vascular metrics at 60dpi were correlated across temporal social behavior (Fig. 4I). Broadly, sham animals had progressively positive correlations between vessel density and average vessel length against absolute partner-interaction (API) across the 60dpi time course but were negatively correlated in TBI mice (Fig. 4I). Total vessel endpoints were strongly correlated (i.e. more vascular fragmentation) with API while maxLFD negatively correlated (Fig. 4I, top row). Both RPI (manual) and RPP (automated) were identical with opposite correlations being observed between groups (Fig. 4I, middle, bottom rows). Interestingly, at 7- and 60dpi, maxLFD had positive correlation with relative partner-interaction for sham but negative correlation with TBI. In sum, TBI mice had opposite correlation trends compared to shams, especially at 60dpi, and the correlation trends across time would suggest that TBI-induced social behavior deficits soon after the injury have predictive potential for long-term vascular decrements.

### Cerebrovascular volumes (CBV) mirror CBF

Cerebral blood volume (CBV) measurements exhibited early post injury declines spanning 14dpi with latent recovery at 30dpi followed by a robust decline at 60dpi (Fig. 4J). CBV in the lesion cortex across time points showed a trending change (*F*(5,104) = 2.20, *p* = 0.059) and were significantly different across injury conditions (sham vs. TBI, *F*(1,104) = 7.27, **p = 0.008, interaction – ns) (Fig. 4K). Shams had higher CBV compared to TBI at baseline (**p = 0.006) and at 14dpi (*p = 0.033). The medial perilesion cortex exhibited significantly different CBV across time (*F*(5,102) = 3.76, **p = 0.004) and injury (*F*(1,102) = 24.2, ***p < 0.001, interaction – ns) with elevated CBV in sham mice compared to TBI at baseline (*p = 0.015), 3 (**p = 0.007), 7 (*p = 0.048), and 14dpi (**p = 0.005) (Fig. 4L). Baseline CBV for sham animals was higher compared to 30dpi (*p = 0.044).

CBV in the lateral perilesional cortex (Fig. 4M) was significantly different across timepoints (*F*(5,101) = 3.68, **p = 0.004) and injury conditions (*F*(1,101) = 6.87, *p = 0.010) with significant interactions (*F*(5,101) = 3.54, **p = 0.005). CBV was initially lower for TBI mice vs. shams at baseline (***p < 0.001) and 3dpi (**p = 0.003) but progressively increased whereas, CBV decreased with time in sham mice (Baseline vs. 3dpi: *p = 0.047, 7dpi: *p = 0.013, 14dpi: **p = 0.002, 30dpi: **p = 0.008, 60dpi: **p = 0.002). At 60dpi TBI mice had elevated CBV compared to 3dpi (**p = 0.002) indicating persistent CBV increases after injury. CBV in regions distant to the injury site, such as the hypothalamus, thalamus and entorhinal cortex exhibited a similar profile with broad decreases in TBI mice compared to shams across most time points (Supplementary Fig. 6).

### Spatiotemporally dispersed effects of TBI

**Fig. 5:**
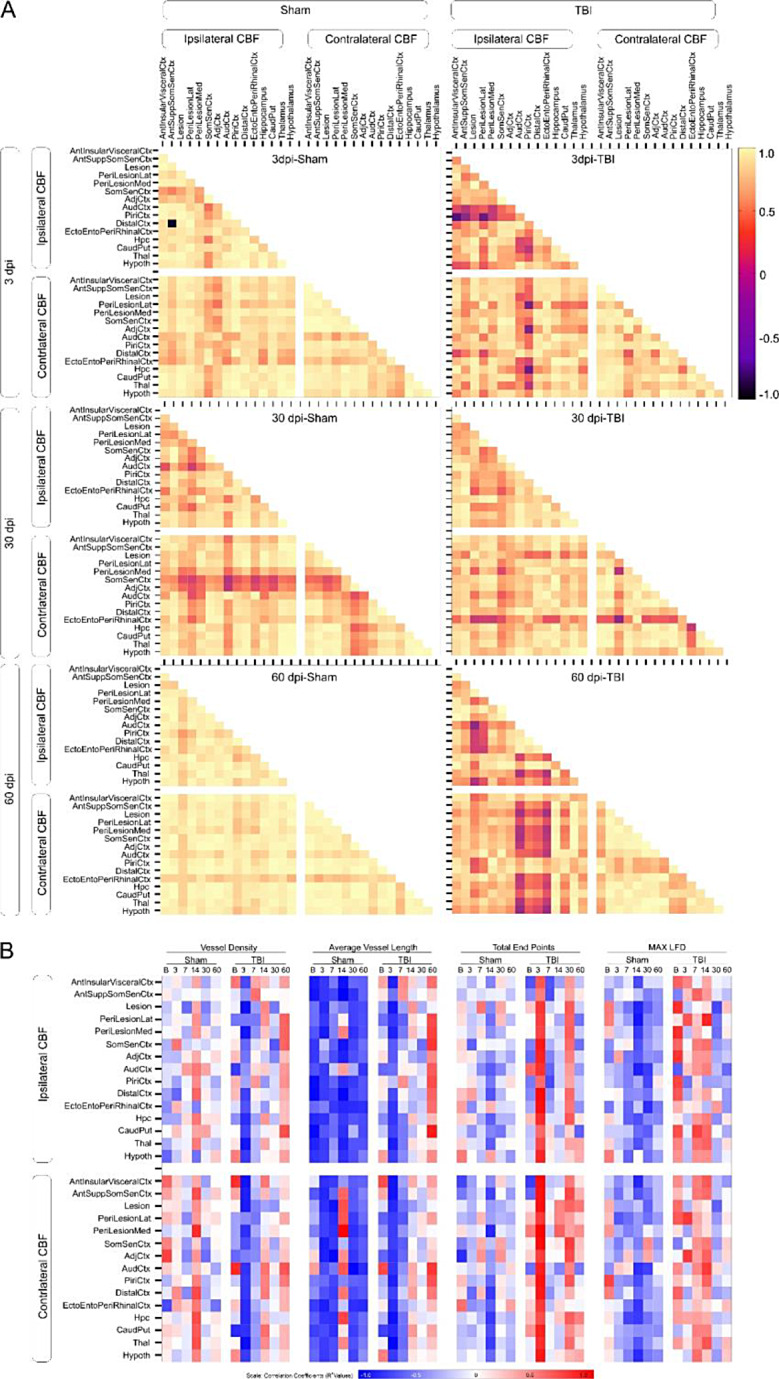
Temporal relationships between cerebral blood flow (CBF) across brain regions. (**A**) Temporal CBF correlation coefficients across brain regions highlight global alterations due to TBI (right panel) resulting in loss of CBF auto-correlations at the injury site at 3dpi that moderately recovers by 30dpi but is greatly perturbed at 60dpi unlike shams (left panel). This dysregulation also spreads to the contralateral hemisphere at 60dpi in TBI mice. (**B**) Temporal CBF correlations to vascular network measures at 60dpi further confirm an initial recovery. However, delayed cerebrovascular structural deficits contributes to the declining brain perfusion. At 60dpi, vessel density in lesion cortex and longitudinal CBF across ipsi- and contralateral brain regions in TBI animals show mostly negative correlations at 3-30dpi followed by positive correlations at 60dpi. In contrast, sham animals show highly positive correlations at 14dpi and low mixed correlations at other time points. Vessel length vs. CBF correlations are negative for sham animals across all timepoints but positive for TBI animals at 60dpi. Total end points and CBF correlations in sham animals also exhibited mostly mixed correlations except 14dpi with negative correlations. Conversely, TBI animals show positive correlations at 3- and 30dpi, negative ipsilateral correlations for 7-60dpi, and negative contralateral correlations at 7dpi, but positive contralateral CBF correlations at 14-60dpi. maxLFD and CBF correlations were negative for sham animals unlike TBI animals with positive correlations at 7-14dpi, negative at 30dpi, and positive again at 60dpi. Abbreviations: Med – Medial, Lat – Lateral, Ctx – Cortex, Hpc – Hippocampus, CaudPut – Caudate putamen, Thal – Thalamus, Hypoth - Hypothalamus

The relationship between ipsi- and contralateral brain regions and their CBF was assessed for temporally related patterns (auto-correlation) and interactions between region and CBF (cross-correlations) (Fig. 5A). Broadly, TBI at 3dpi resulted in lower autocorrelations of CBF to ipsilateral compared to contralateral brain regions (Fig. 5A, top panel), which contrasts to the uniform bilateral correlations in shams. Early ipsilateral CBF dysregulation in TBI animals recovered by 30dpi (Fig. 5A, middle panel), which coincides with vascular recovery [25]. However, at 60dpi when both CBF and vessel density are reduced, CBF auto-correlations across multiple brain regions are dramatically reduced (Fig. 5A, bottom panel) in stark contrast to sham mice that exhibit strong bilateral CBF auto-correlations, as would be expected in healthy mice. These findings confirm the prolonged secondary consequences of TBI on blood flow across broad portions of the brain, including those distant from the injury and mirror angioarchitecture.

We next probed if early CBF dynamics (3-30dpi) predict the imminent secondary vascular damage late after injury (60dpi). Correlations between longitudinal CBF (Baseline–60dpi) and vascular metrics measured within lesion cortex at 60dpi (Fig. 5B) demonstrate distinct longitudinal correlation patterns in sham and TBI mice. Roughly, in TBI mice the correlations suggest an initial negative correlation(s) between vessel density and length that increasingly, with time become strongly correlated by 60dpi. These observations are opposite in vessel endpoints and vascular complexity measures (LFD). Thus, early (3-7dpi) blood flow and vascular disruption are not synchronized whereas the low CBF and loss of the vascular network are tightly correlated at 60dpi. In summary, CBF measures after TBI may reflect altered vascular morphology.

### Neurovascular function corresponds to social behavior decrements

**Fig. 6:**
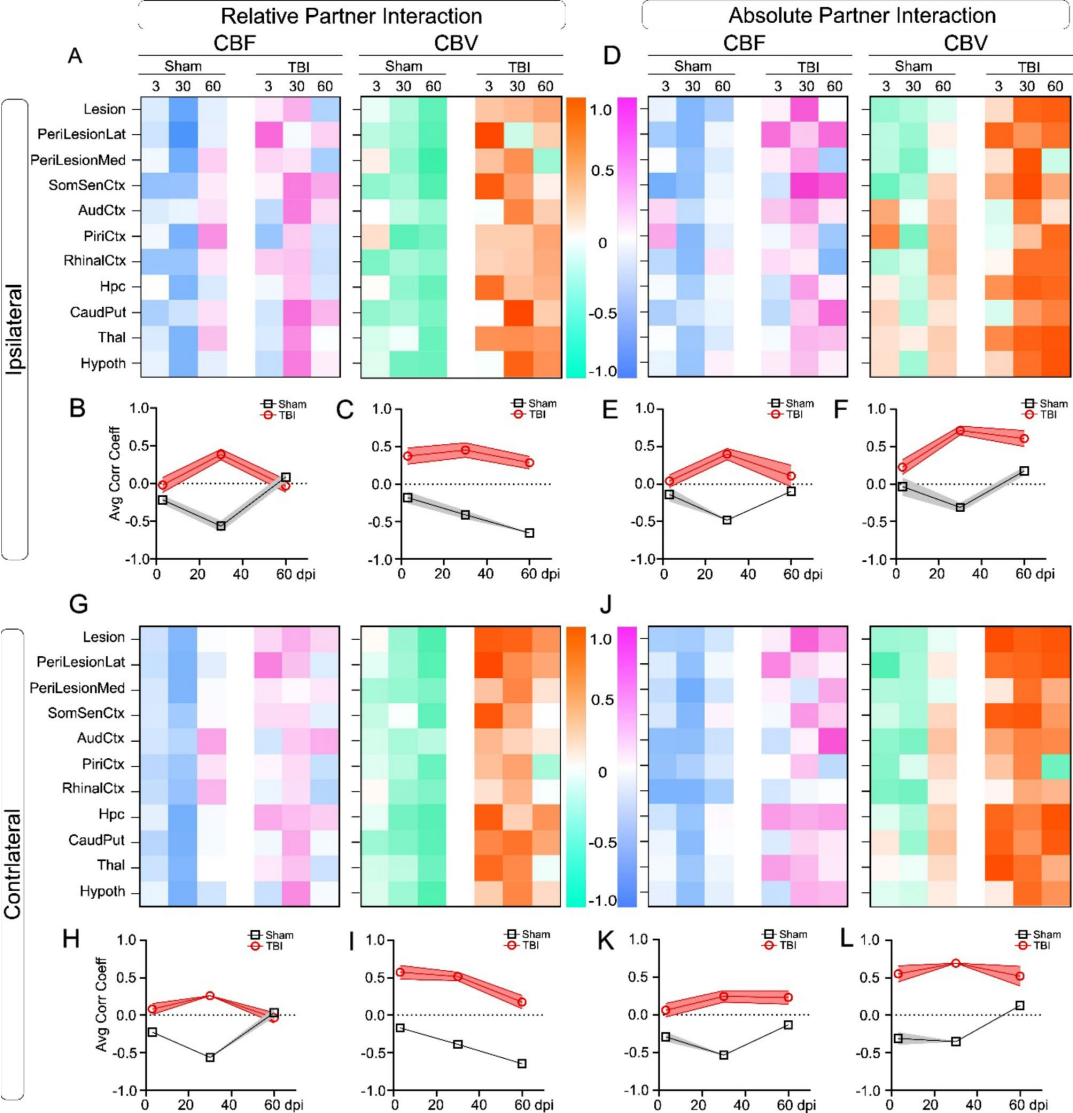
Neurovascular physiology in social behavior associated brain regions. (**A**-**C**) Correlation coefficients of relative partner interaction (RPI) to CBF and CBV across ipsilateral social behavior associated brain regions in TBI and sham mice at 3, 30, and 60dpi (columns). Regionally averaged correlation coefficients are illustrated in B, C. (**B**) TBI animals exhibit the opposite correlations at 30dpi with strong positive correlations unlike negative correlations in shams for RPI vs. CBF. (**C**) In TBI mice RPI vs. CBV show strong correlations across time in contrast to shams having negative correlations with behavior. (**D**) Correlations of absolute partner interaction (API) with ipsilateral CBF and CBV exhibit a virtually identical (compared to RPI) set of regional correlations between sham and TBI animals. (**E**) API vs. CBF correlations exhibit identical trends as RPI vs. CBF correlations for both sham and TBI animals with the largest divergence at 30dpi. (**F**) API vs. CBV correlations are elevated in TBI mice compared to reduced strength correlation patterns in shams. (**G**) Correlation coefficients of RPI vs. CBF and CBV across contralateral brain regions behavior have the same temporal patterns as the ipsilateral brain regions. (**H**) Contralateral CBF vs. RPI at 30dpi have increased correlations in TBI mice but reduced in shams with no overt differences at 3 or 60dpi. (**I**) As in the ipsilateral regions, RPI vs. CBV correlations were strongly positive in TBI mice but greatly reduced temporally in shams **(J)** API correlation to contralateral CBF and CBV were strong across all regions in TBI but not shams. **(K)** API vs. CBF correlations in shams were negative compared to TBI animals showing stable moderate positive correlations. **(L)** API vs. CBV correlation patterns were strongly positive for TBI mice but predominately negative in sham animals. Abbreviations: Med – Medial, Lat – Lateral, Ctx – Cortex, Hpc – Hippocampus, CaudPut – Caudate putamen, Thal – Thalamus, Hypoth - Hypothalamus

To capture associations between cerebrovascular function (CBF, CBV) and social behavior longitudinally after TBI, we undertook correlations across bilateral regions. This approach demonstrated strong linkage of RPI to ipsi- and contralateral cerebrovascular decrements across social behavior related brain regions (ipsilateral, Fig. 6A-C; contralateral, Fig. 6G-I). In general, the correlation matrices, as evident in similar heatmaps between ispi- and contralateral regions were remarkably consistent. When all regional correlations were averaged, we observed distinct signatures that separated TBI mice from shams (Fig. 6B-F, H-L). TBI induced ipsilateral CBF and CBV changes which strongly correlated with RPI and API but was negatively correlated in sham mice. Compared to CBF, the CBV changes exhibited the strongest correlations on both ipsi- and contralateral brain regions (e.g. Figure 6C, I). We also note virtually identical correlations across all social behavior metrics (Supplementary Fig. 7). In summary, CBF vs. RPI/API correlations increase from 3- 30dpi but then decline by 60dpi while sham animals show opposite trajectories (Fig. 6B, E). CBV correlation profiles with RPI/API have progressive negative correlations in shams whereas TBI mice have persistent positive correlations (Fig. 6C, F). Similar correlational directionality was observed between CBF/CBV and RPI/API in contralateral brain regions (Fig. 6G-L). Profiles showing API correlated with ipsilateral (Fig. 6D-F) and contralateral (Fig. 6J-L). Hence, early correlations between behavior and CBF predict long-term physiological deficits.

## Discussion

We investigated longitudinal and dynamic evolution of behavior and vascular physiology across 2-months post-TBI (Fig. 1). Our study is the first preclinical TBI study to demonstrate reduced social preference between familiar cage-mates when vascular functions decline. The key findings are: (i) a late decline (60dpi) in sociability of TBI animals while motor and exploratory behavior recover; (ii) longitudinal CBF (Figs. 2 and 3) identifying a novel multiphasic recovery profile with delayed 60dpi global decline in perfusion that also encompasses brain regions processing social behavior; (iii) angioarchitecture exhibits vascular damage at 60dpi, including reduced vessel density and increased fragmentation (Fig. 4); (iv) vascular and social behavior correlations displayed pronounced negative correlations early after TBI but progressively become positively correlated by 60dpi; iv) regional correlations confirmed dynamic bilateral CBF changes across the brain after a unilateral TBI, (Fig. 5) that becomes predominately perturbed ipsilaterally; and (v) unique correlation patterns between behavior and vascular physiology emerge in TBI animals (Fig. 6). In summary, our study for the first-time links how cerebrovascular physiology may contribute to the decline in social interactions after TBI in male mice. Our findings also suggest that monitoring social behavior early after brain injury may predict long-term neurovascular damage, providing a putative avenue for therapeutic interventions.

Neuropsychological disabilities, including sociability issues are being increasingly recognized after TBI in adults [47] and children [48]. Subjects often exhibit the inability to recognize affective facial expressions, [49, 50] impaired verbal and non-verbal communication, [9] and diminished empathy, [51] culminating into decreased time spent with friends and families. Interestingly, brain regions regulating social behavior are similar between rodents and primates [52, 53]. Feature content of socially transferred food preference memory is known to decline after systems consolidation, [40] and social isolation elicits similar pathophysiology in healthy rodents as in humans [54]. A few studies, in preclinical models of TBI, have explored social dominance and interaction behavior [20, 55]. Our observation of behavioral recovery at 30dpi corroborates a recent study that showed lack of social deficits at 30dpi in mice with repeated mTBI [56]. Consistent with the previous literature we also found TBI animals cover longer distances than the shams, at higher relative speed [57]. Preferentially exploring and frequenting the center of the open-field arena suggests an inclination for taking risks, a well-documented behavior observed in rodents and human subjects after TBI [58–60]. However, long-term social isolation among familiar individuals post-TBI has not been studied.

In chronic TBI, cognitive impairments and social isolation often precede the debilitating prolonged consequences of injury among human subjects that parallel Alzheimer’s-related signatures, [61] depression, and suicidal tendencies [62]. Our novel approach captures the long-term social interaction deficits between familiar mice as observed in head-injured human subjects [63, 64]. In the classic Crawley’s 3-chamber test, [37, 65] a test mouse interacts with a combination of un-familiar mice and a novel object where the social performance reflects combined effect of novel entity and neophobia [66]. An adult frontal TBI model showed increased preference for familiar mice in 3-chamber task, however, longitudinal transition of social behavior in same animals was not investigated [16]. Our temporal study demonstrated, in TBI mice, no initial sociability deficits but at chronic time points the emergence of a decline in voluntary preference of a known animal i.e. their cage-mate partner. This decrement in sociability of TBI mice was accompanied by decreased anxiety and increased exploration consistent with increased risk behavior, as previously reported [67]. 

Numerous studies have sought to link altered behavior to inflammation [68] and neuronal cell death [69] as underlying mechanisms. Surprisingly, there are limited investigations into how cerebrovascular morphology and function are linked after TBI to behavioral deficits. A recent study in TBI subjects found significant associations at ~ 2yrs post injury between decreased perfusion and psychoemotional outcomes (i.e. anxiety) [70]. Neurovascular coupling implies a link between task-evoked cellular metabolic demands and blood flow to the activated brain region [71]. Social recognition associated cellular networks require protein synthesis and includes cAMP responsive element-binding protein (CREB) for transcriptional consolidation [72, 73]. Adult human TBI leads to resting hypoperfusion in many task-related brain regions years after injury and is similar to that reported in TBI rodent models [31, 74]. In our study we also found early hypoperfusion that recovered by 30dpi but then rapidly declined in virtually every brain region. Interestingly at 60 dpi, we found reduced CBF in dorsal hippocampus (dHpC) whose subregions are necessary for spatial exploration (CA1) and social interaction (CA2) [75]. Reduction in interaction with familiar in comparison to novel conspecifics in chronic-TBI could be driven by corticotrophin signaling from PFC to lateral septum [76]. Two months after the injury, reduction in intrinsic excitability and decreased synaptic output of somatostatin neurons in layer V of orbito-frontal cortex has been reported [77], consistent with long-term behavioral deficits as shown in ours and other studies [78, 79]. Such deficits could arise from the TBI-associated neuronal damage in cortex, dorsal hippocampus and thalamus and future studies can shed more light on the neural underpinnings of similar TBI comorbidities. Along with this rationale, our data captures a delayed decline in overall neurovascular functional health and maintenance in brain regions associated with social behavior. Our results show reduced regional CBF post TBI with a novel multiphasic pattern of intermittent recovery at 7 and 30dpi, worsening of blood flow at 14dpi and a delayed decline at 60dpi. Interestingly, we observe similar longitudinal profiles for RPP and CBF measurements in social behavior relevant brain regions.

Our results are consistent with previous studies showing acute and chronic hypoperfusion and behavior deficits in both clinical and preclinical TBI [57, 80–82]. Several studies have demonstrated recovery of behavioral performance after TBI in rats, [83] mice, [57, 84] and humans [85]. The primary finding of many studies is that social, anxiety and cognitive behavioral domains worsen with increasing time. A similar progressive decline in brain perfusion long-term has been reported. Human studies report CBF recovery by 3 weeks post injury, with linkage to improved neurological outcomes [86]. Grohn and colleagues noted biphasic hypoperfusion with transient recovery followed by a second hypoperfusion epoch over two weeks after TBI, that relates to changes in vascular density [87]. Others have also noted protracted CBF reduction in rodents that has been shown to last up to 1 year post TBI [88]. Our current study and previous observations demonstrated perfusion recovery at 30dpi [25] that was reflected by recovery of vascular density. It is important to note that brain-wide circuit reorganization has been well documented after TBI that likely impacts behavioral outcomes [89]. In focal ischemic injury there is a dissociation between CBF and neuronal activity that could impact behavior [90]. In our study the consistent decrement in CBF at 60dpi relates to reduced vascular density and complexity which may exacerbate behavioral deficits and may impact regional connectivity.

In support of our study, previous findings noted that focal TBI elicits longitudinal global cerebrovascular deficits underlying large-scale brain network effects, likely leading to protracted social deficits [91]. Bilateral CBF reductions, similar to our observations, have been seen in human mTBI subjects for prefrontal cortex, putamen, and hippocampus, while reduced CBF in cortex and caudate putamen is associated with depressive symptoms, and in hippocampus with anxiety [70]. Consistent with the previous human studies, we also observe thalamic pathophysiology after mTBI [92]. Decreased thalamic dendrite complexity in rats also showed recovery by 4-weeks post mTBI, [93] corroborated by corresponding vascular and functional perfusion recovery in our study that subsequently collapses by 60dpi.

TBI results in immediate damage to focal and distant cerebrovascular morphology that then partially recovers [23, 25, 94]. The subsequent secondary cellular and molecular cascades after moderate to severe TBI result in long-term deficits including hemorrhage, edema, reduced CBF, vasospasms, blood-brain disruptions, coagulopathy, and chronic inflammation [95–97]. Surprisingly, we observed a second period of vascular loss at 60dpi despite vascular recovery by 30dpi [25]. The late diminished vessel characteristics mirrored reduced CBF virtually across all brain regions compared to shams. Thus, initial vascular recovery is transient and is not well integrated within the brain parenchyma as stable neurovascular units [98, 99]. Structural vascular abnormalities in human TBI subjects exhibit microvessels with flattened, reduced lumina and longitudinal folds in the pial, cortical, and capillary zones [26, 100]. In lateral fluid percussion rodent models there also is microvascular recovery which does not mirror healthy control vasculature [87, 101]. As noted previously, we observed identical recovery profiles after TBI [25] which then rapidly degrade by 60dpi. Vascular density was also increased at 14dpi after repeated TBI concomitant with diminished CBF, cerebrovascular reactivity, and neuronal activity [102]. In adult and pediatric human subjects after TBI, CBF and CBV decline [47, 103]. Broadly, TBI in clinical and preclinical studies suggest that dynamic vascular density alterations lead to chronic reductions in brain responsivity and perfusion.

It is noteworthy that there are regional variations, particularly in correlative preclinical studies. For example, hippocampal vessel density does not vary with declining CBF whereas increases in CBF and vessel density were reported in ipsilateral thalamus 8-months after TBI [28, 31, 47]. The authors reported that poor spatial exploration performance correlated with increased thalamic vessel density. Griffiths and colleagues reported no changes in cortical or hippocampal CBF or CBV 6-months after mild TBI despite cognitive decrements [104]. Similar findings have been reported in individuals with mild [70]and in moderate severe TBI [105]. 

Our correlations measure the interdependent blood flow across brain regions highlighting bilateral effects unique to TBI animals. Specifically, global blood flow correlations were reduced at 3dpi with recovery by 30dpi followed by a dramatic decline at 60dpi. The correlations of longitudinal behavior to 60dpi vascular metrics provided an early-stage behavioral marker to predict the imminent long term neurovascular damage from TBI. Finally, our correlations between social behavior, blood flow and blood volume in brain regions exhibited unique patterns for TBI animals at 3, 30- and 60dpi. Similar behavioral correlations to CBF were found in mTBI [104]. 

The strengths of our study are, (i) longitudinal in vivo assessments of behavior alongside cerebrovascular function after TBI which provide a continuous view of how recovery is modulated; (ii) behavioral tests across multiple domains (motor, exploratory, social) but with novel social preference for familiar cage mates, an observation reported in human subjects yet underexplored in preclinical models; (iii) our extended observation window to 2-months post-TBI which is equivalent to ~ 7 years in humans [106], (iv) novel multiphasic global evolution of brain perfusion after injury in the same subjects; (v) novel CBF autocorrelations across whole brain regions demonstrating remission of cerebrovascular pathophysiology at 30dpi but recurrence at 60dpi; (vi) the loss of structural vascular networks near the impact site, coupled with spatially dispersed secondary chronic effects underlie CBF and social behavior deficits; and vi) correlations across behavior and cerebrovascular physiology provide a predictive assessment of imminent long-term pathophysiology underlying TBI comorbidities.

Several mechanisms that may be responsible for the findings reported herein; moreover, these mechanisms very likely act synergistically to elicit the decrement in vascular function. We speculate that the decline in morphology and function is multi-faceted: (1) Recent studies have noted vascular pruning is accompanied by microglial clearing of endothelial cells [107], (2) microglia modulate blood-brain-barrier dysfunction in TBI and are linked to vascular leakage [108], (3) Systemic inflammation evokes microglia appear to initially maintain the BBB but sustained inflammation results in astrocyte endfeet loss and impaired BBB [109], (4) Microglia are vasoregulatory and their transcriptome express vasoreactive genes [110], (5) Pericytes and microglia are intimately integrated in capillary function and loss of this association contributes to vascular dysfunction [111], and (6) While the current study focused exclusively on the evolution of vascular perturbations and their impact on social behaviors, it would be important to directly assess regional connectivity between regions implicated in social behavior. Diffusion MRI or resting state MRI could be and have been used to assess changes in regional connectivity after TBI [112]. Each of these potential mechanisms are likely involved in the temporal blood flow trajectory we report. Clearly, additional studies are required to dissect these mechanisms in more detail.

There are several limitations of our current study. They include: (i) absence of female mice perfusion and behavioral data. Female gender is underrepresented in clinical and most pre-clinical research studies and emerging studies suggest that women of the same age group (compared to men) are more susceptible to adverse consequences of TBI [113]. To fill this gap we are currently investigating the long term physiological and behavioral pathology post-TBI in female mice; (ii) some of the variance in our measurements can be attributed to the modest number of replicates (*n* = 6–8/grp/time) but exhibited sufficient statistical power particularly in light of our longitudinal assessments; (iii) limited anatomical resolution from the perfusion-weighted MRI measurements. Due to the rapid acquisition techniques in-plane resolution was 250 μm/pixel which provide sufficient resolution for regional brain assessments using manual segmentations based on the Allen brain atlas [114]. Future studies will address these limitations by combining high-resolution optical with magnetic resonance imaging. However, MRI does provide a powerful non-invasive and longitudinal global assessment of pathophysiology that is not feasible with other techniques; (iv) baseline CBF variations between sham and TBI groups before injury were noted in some but were not different for regions involved in social behavior (hippocampus, piriform, auditory, rhinal cortices); and, (v) use of isoflurane anesthesia is known to result in a dose-dependent biphasic alterations in cerebrovascular flow (vasoconstriction and vasodilation) [115, 116] and may impact the variance we observed in sham mice. Regional sensitivity to vascular responses has also been reported with sub-cortical regions being more affected by isoflurane [117], although the effects of repeated anesthetic exposure have not been reported.

## Conclusions and future directions

In conclusion, our study for the first time demonstrates the possible cerebrovascular underpinnings of emerging social behavioral deficits after chronic TBI. Social interactions among familiar mice long after a TBI were reduced with concurrent longitudinal physiological reductions in CBF and CBV. A steep decline in CBF at 60dpi in social behavior related brain regions was observed in hippocampus and rhinal cortex. The loss of angioarchitecture at 60dpi provides the basis for precipitous declines in CBF and social behavior. Further, our correlations point to broad linkage between impairments in vascular metrics, CBF, CBV, and social behavior metrics. We suggest that such correlations may have predictive value for obtaining early estimates of long-term damage, and potentially informing the optimal treatments. In addition to pharmacological interventions, enriched environments with monitored exercise [118–120] and virtual environments are deemed helpful during chronic TBI recovery in rodents [121] and humans [122, 123], including virtual social networks [124]. Future investigations, in addition to assessing influence of sex, should investigate how vascular smooth muscle attributes are modified by TBI [125] and how pericytes regulate blood flow [126]. Finally, chronic TBI sequelae such as BBB dysfunction, TGFβ signaling, and neuroinflammation also contribute to the long-term effects of injury. While our study in a rodent preclinical model hints at the linkage between neuropsychological outcomes modulated by brain perfusion, continued investigations are needed to improve our understanding of the longitudinal implications of TBI and how we might best intervene to improve patient outcomes.

### Electronic supplementary material

Below is the link to the electronic supplementary material.

Supplementary Material 1

## Data Availability

Data is available at a reasonable request from the corresponding author.
